# Water Storage Trends in High Mountain Asia

**DOI:** 10.3389/feart.2019.00235

**Published:** 2019-09-26

**Authors:** Bryant D. Loomis, Alexandra S. Richey, Anthony A. Arendt, Ravi Appana, Y.-J. C. Deweese, Bart A. Forman, Sujay V. Kumar, Terence J. Sabaka, David E. Shean

**Affiliations:** 1 Geodesy and Geophysics Laboratory, NASA Goddard Space Flight Center, Greenbelt, MD, United States; 2 Department of Civil and Environmental Engineering, Washington State University, Pullman, WA, United States; 3 Applied Physics Laboratory, University of Washington, Seattle, WA, United States; 4 Civil and Environmental Engineering, University of Maryland, College Park, MD, United States; 5 Hydrological Sciences Laboratory, NASA Goddard Space Flight Center, Greenbelt, MD, United States; 6 Civil and Environmental Engineering, University of Washington, Seattle, WA, United States

**Keywords:** terrestrial water storage, High Mountain Asia, GRACE mascons, glacier mass balance, groundwater, land information system

## Abstract

Changes in terrestrial water storage (TWS) in High Mountain Asia (HMA) could have major societal impacts, as the region’s large reservoirs of glaciers, snow, and groundwater provide a freshwater source to more than one billion people. We seek to quantify and close the budget of secular changes in TWS over the span of the GRACE satellite mission (2003–2016). To assess the TWS trend budget we consider a new high-resolution mass trend product determined directly from GRACE L1B data, glacier mass balance derived from Digital Elevation Models (DEMs), groundwater variability determined from confined and unconfined well observations, and terrestrial water budget estimates from a suite of land surface model simulations with the NASA Land Information System (LIS). This effort is successful at closing the aggregated TWS trend budget over the entire HMA region, the glaciated portion of HMA, and the Indus and Ganges basins, where the full-region trends are primarily due to the glacier mass balance and groundwater signals. Additionally, we investigate the closure of TWS trends at individual 1-arc-degree mascons (area ≈12,000 km^2^); a significant improvement in spatial resolution over previous analyses of GRACE-derived trends. This mascon-level analysis reveals locations where the TWS trends are well-explained by the independent datasets, as well as regions where they are not; identifying specific geographic areas where additional data and model improvements are needed. The accurate characterization of total TWS trends and its components presented here is critical to understanding the complex dynamics of the region, and is a necessary step toward projecting future water mass changes in HMA.

## 1. INTRODUCTION

Secular changes in High Mountain Asia (HMA) terrestrial water storage (TWS) can modify global mean sea level (Reager et al., 2016) and affect the availability of freshwater for the more than one billion people living in the region (Wester et al., 2018; Pritchard, 2019), motivating the accurate determination of TWS trends and the partitioning of individual components. The hydrology of the HMA region is complex due to the multiple cryospheric sources of runoff (snow, glacier and permafrost melting), the influence of complex topography and monsoon dynamics on precipitation distribution, and rapidly shifting patterns in irrigation practices. Existing models show high variability in runoff composition for each of the major HMA river basins and provide estimates of future trends in runoff in a changing climate (Lutz et al., 2014; Armstrong et al., 2018); however, there are few independent observations available to calibrate and validate these simulations.

The launch of the Gravity Recovery and Climate Experiment (GRACE) in March 2002 revolutionized the ability to monitor TWS on a global scale to a spatial resolution of 300–500 km (Gaussian smoothing half-radius) (Wahr et al., 1998; Tapley et al., 2004; Luthcke et al., 2013). While GRACE is extremely valuable for its unique ability to recover the full TWS signal, its standard monthly products are somewhat limited in their application due to relatively low spatial resolution as compared to other remote sensing measurements, *in situ* observations, and model outputs. A number of previous studies have applied the monthly GRACE data products for determining TWS changes in HMA at large basin scales (length ≥500 km; area ≥250,000 km^2^), with some comparisons made to individual TWS components (Matsuo and Heki, 2010; Moiwo et al., 2011; Jacob et al., 2012; Rodell et al., 2018; Scanlon et al., 2018). Studies that attempt to disaggregate GRACE TWS into individual components take two approaches, either isolating the residual of interest by using independent data and models to represent the remaining water budget components or by assimilating GRACE into land surface models (Frappart and Ramillien, 2018). The first method propagates error from the independent data or models into the residual of interest. The second method can inaccurately distribute mass change within the model if the model is missing components that are included in the GRACE signal. While the assimilation approach has successfully reduced groundwater uncertainties in certain areas, it performs less well in regions dominated by human dynamics that are not capture in the model (Frappart and Ramillien, 2018). Scanlon et al. (2018) recently demonstrated that land surface models are not able to match decadal trends in TWS as seen by GRACE in large global river basins, indicating a clear need to identify the source of discrepancies from the models to improve projections of future water storage change. To date, no HMA study we are aware of has demonstrated the successful closure of the individual TWS trend components with the GRACE-derived total, or attempted to extract sub-basin scale (<300 km) mass trends with GRACE.

**FIGURE 1 ∣:**
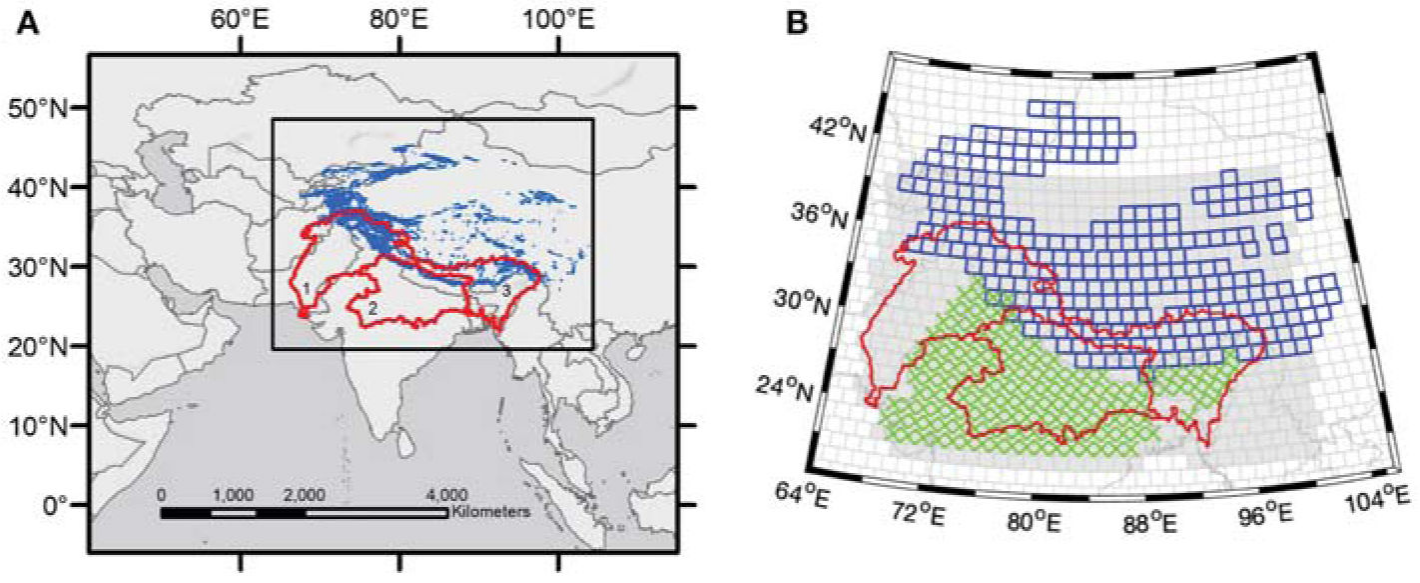
**(A)** The High Mountain Asia region, including glacier extent from Pfeffer et al. (2014) (blue) and the major basins in the region (red): 1-Indus, 2-Ganges, and 3-Brahmaputra. **(B)** Zoom-in of the HMA region showing the data distribution of the Land Information System (shaded gray), groundwater measurements (green X’s), and geodetic glacier mass balance observations (blue boxes). The visible cells correspond to the HMA subset of the global 1-arc-degree NASA GSFC mascon product.

In this work we examine the TWS trend budget in an attempt to close the budget for the full HMA region, the glaciated sub-region, the Indus, Ganges, and Brahmaputra basins (Figure 1), and at sub-basin spatial scales within HMA that correspond to the NASA Goddard Space Flight Center (GSFC) 1-arc-degree GRACE mascons (length ≈110 km; area ≈12,000 km^2^) (Loomis et al., 2019). We present several important advancements toward understanding secular changes in HMA TWS with a new GRACE mascon product, *in situ* data, and innovative methods applied in the recovery of individual TWS components derived from independent studies. We present the results of a new GSFC global mascon product that directly estimates regression model parameters from the GRACE Level 1B measurements (referred to hereafter as “L1B regression mascons”) from which a trend (i.e., regression slope) may be inferred. These GRACE-only trend estimates approach a spatial resolution of ~110 km and achieve significant improvements in the magnitude of the recovered signal as compared to trends determined from the monthly GRACE products. This new product also includes a rigorous assessment of the uncertainties, which accounts for the solution bias that results from the regularized estimation of the mascon parameters. This new L1B regression product facilitates a comparative analysis between GRACE-derived TWS and other HMA models and data sets at a higher spatial resolution than was previously possible.

In an effort to partition the GRACE-derived HMA TWS trends into their major components, we consider the following data sets: (1) new geodetic glacier mass balance estimates from ~36,000 Digital Elevation Models (DEMs) (Shean et al., submitted); (2) groundwater storage changes inferred from well measurements separated into confined and unconfined systems; and (3) model outputs of eight different Land Information System (LIS; Kumar et al., 2006) runs that employed two different Land Surface Models (LSMs) using four different sets of meteorological boundary conditions (Kumar et al., 2012; Yoon et al., 2019). The geographic distribution of these different data products is shown in Figure 1B. With a rigorous characterization of the GRACE mascon uncertainties that accounts for both noise and bias, we are able to identify basins and individual mascons where closure is, and is not, achieved. When budget closure is achieved we assume we have successfully identified the primary driver(s) of the TWS trends, while lack of closure highlights the geographic locations in HMA where additional data or future model development is needed.

## 2. DATA AND METHODS

### 2.1. GRACE

#### 2.1.1. Monthly Mascons

The standard Level 2 time-variable gravity product provided by the GRACE project is a series of unregularized spherical harmonic coefficients estimated at monthly time steps (Wahr et al., 1998). Due to larger noise at the higher spherical harmonic degrees (smaller spatial scales), it is necessary to apply post-process filtering to retrieve geophysically meaningful global maps or regional time series of mass change (Wahr et al., 1998; Wouters et al., 2014). Many different filters and methods have been developed over the course of the mission, where the selected approach can significantly impact the results. For example, Jacob et al. (2012) determined a HMA glacier trend of −4 ± 20 Gt yr^−1^ while Matsuo and Heki (2010) reported −47 ± 12 Gt yr^−1^ over approximately the same time period using the same Level 2 product but different post-processing techniques. More recently, regularized global mass concentration solutions (mascons) have emerged as a preferred time-variable gravity product for many researchers, with different monthly products provided by NASA GSFC (Luthcke et al., 2013; Loomis et al., 2019), the Jet Propulsion Laboratory (JPL) (Watkins et al., 2015), and the University of Texas Center for Space Research (CSR) (Save et al., 2016). Regularized mascon estimation is a more optimal approach for improving the solution signal-to-noise ratio because the time-variable gravity parameters are directly estimated from the Level 1B data while fully accounting for the noise and signal covariance matrices (Sabaka et al., 2010) thereby eliminating the need for post-processing. Another advantage of the mascon approach is the ability to introduce constraint regions that significantly mitigate signal leakage across constraint boundaries (e.g., land and ocean), effectively increasing the spatial resolution at these boundaries. It is important to note, however, that the fundamental resolution of the mascon solutions (300–500 km) is the same as the spherical harmonics within a constraint region (Luthcke et al., 2013). In the analysis of basin-scale TWS trends we present results for the GSFC, JPL, and CSR monthly mascon products along with their goodness-of-fit uncertainties, which do not account for signal leakage. We note that all GRACE results presented in this work have had the ICE-6G_D glacial isostatic adjustment (GIA) model removed (Peltier et al., 2018).

#### 2.1.2. L1B Regression Mascons

A fundamental challenge of working with GRACE data and its application to understanding TWS variability is the limited spatial resolution of the GRACE data products. The spatial resolution of the GRACE products is determined by a complex combination of factors including the accuracy of the inter-satellite instrument, the spatiotemporal sampling of the ground tracks, and errors in the atmospheric and ocean dealiasing models applied in the processing in an effort to remove those high-frequency signals from the monthly gravity solutions. Throughout the duration of the GRACE mission, various static (mean) spherical harmonic gravity fields have been estimated to much higher spatial resolution than is possible for the time-variable monthly fields. A fundamental trade-off exists between the spatial and temporal resolution of GRACE-derived gravity estimates, where increased spatial resolution is achieved with the accumulation of multiple years of data (Pail et al., 2010), and sub-monthly solutions have lower spatial resolution than the monthly products (Croteau, 2019).

**FIGURE 2 ∣:**
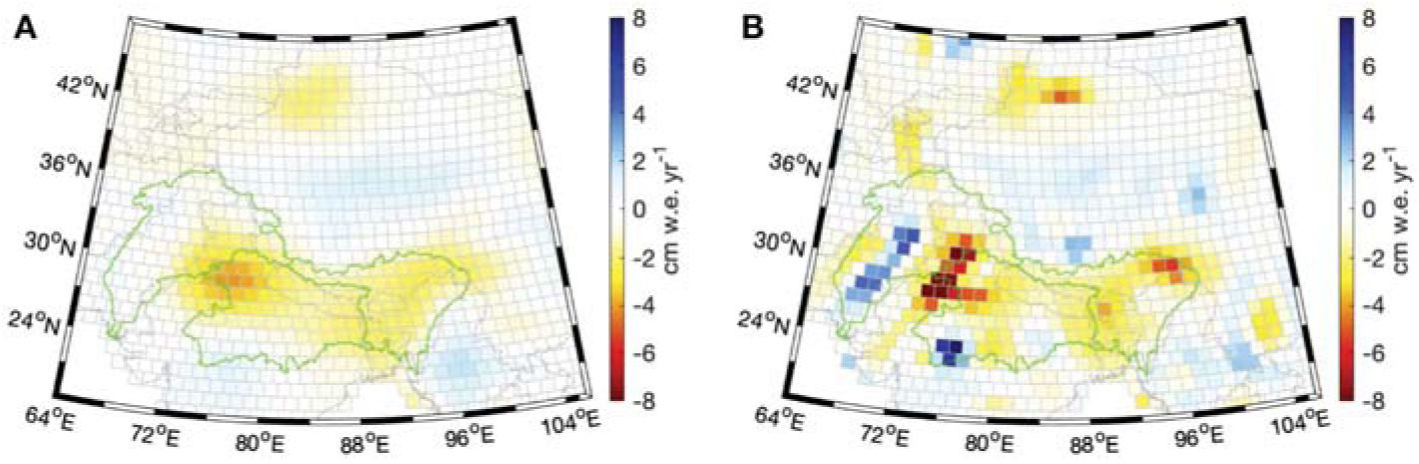
GRACE mass trends in terms of centimeter water equivalent per year (cm w.e. yr^−1^), as determined from **(A)** the GSFC monthly mascon solution, and **(B)** the GSFC L1B regression solution. Trends are computed for January 2003–July 2016.

Several of these static gravity fields determined from GRACE, such as the GOCO and EIGEN spherical harmonic models (Pail et al., 2010; Rudenko et al., 2014), co-estimate time-variable components such as a trend and annual signal along with the mean component. These “static” spherical harmonic gravity products recover the time-variable model components to a higher spatial resolution than is possible with a single month of data. Recognizing the benefit of regularized mascon estimation, NASA GSFC has recently expanded this same concept by estimating a regression model for each of its 41,168 1-arc-degree mascons using more than 13 years of GRACE data (January 2003–July 2016). The product that is discussed in this work co-estimates a bias, trend, and annual signal for a total of four parameters for each mascon. Additional model parameters can be estimated if desired and the relevant term for this work is the recovered trend. Figure 2 clearly demonstrates the improved spatial resolution and signal recovery for the regression product as compared to estimating the trend from the series of monthly estimates. This new, regularized L1B regression mascon product has improved signal recovery as compared to the multi-year spherical harmonic estimates, and presents an opportunity to study GRACE-derived TWS trends to a much higher spatial resolution than was previously possible, allowing for a more direct comparison with model output and *in situ* observations.

To understand the improvement of the L1B regression mascon products over the time series derived from explicit GSFC monthly mascon solutions, consider the adjustment to the mascons for the *j*th month, ${\hat{m}}_{j}$, which are assumed to be static within the month, such that

(1) $${\hat{m}}_{j}={\left(A_{j}^{T}W_{j}A_{j}+P_{j}\right)}^{-1}A_{j}^{T}W_{j}d_{j},$$

where **A***_j_* is the design matrix that relates the L1B inter-satellite observations to the mascons, **W***_j_* is the inverse of the data noise covariance, **P***_j_* is the inverse of the signal noise covariance, and **d***_j_* is the vector of inter-satellite residuals. This expression is commonly referred to as Tikhonov regularization (Tikhonov, 1963). Note that in Equation (1) we have assumed an *a priori* mascon state of zero. In the explicit method, the time series of the *k*^th^ mascon assembled from the *j*=1, . . . , *N*_*t*_, estimates, ${\hat{m}}_{k}^{T}=\left[{\hat{m}}_{1,k}\ldots {\hat{m}}_{j,k}\ldots {\hat{m}}_{N_{t},k}\right]$, where *N_t_* is the number of months for which estimates are available, is fit with *N_f_* temporal basis functions whose multipliers, ${\hat{x}}_{k}$, are estimated as

(2) $${\hat{x}}_{k}={\left(F^{T}F\right)}^{-1}F^{T}{\hat{m}}_{k},$$

where, in the case of basis functions such as bias, trend, and an annual sinusoid, *N_f_*=4 and the elements of $F\in R^{N_{t}\times N_{f}}$ are given by

(3) $$F_{j1}=1,\;F_{j2}=t_{j},\;F_{j3}=\cos\;2\pi \;t_{j},\;F_{j4}=\sin\;2\pi \;t_{j},$$

with *t_j_* rendered in units of years. If we collect the *N_s_*=41,168 bias, trend, cosine, and sine multipliers into the vectors, ${\hat{x}}_{b}$, ${\hat{x}}_{t}$, ${\hat{x}}_{c}$, and ${\hat{x}}_{s}$, respectively, then a simultaneous inversion for all multipliers in the explicit method may be expressed as

(4) $$\hat{x}=\left[{\left(F^{T}F\right)}^{-1}F^{T}⊗I\right]\hat{m},$$

where ${\hat{x}}^{T}=\left[{\hat{x}}_{b}^{T}{\hat{x}}_{t}^{T}{\hat{x}}_{c}^{T}{\hat{x}}_{s}^{T}\right]$, ${\hat{m}}^{T}=\left[{\hat{m}}_{1}^{T}\ldots {\hat{m}}_{j}^{T}\ldots {\hat{m}}_{N_{t}}^{T}\right]$, $I\in R^{N_{s}\times N_{s}}$ is an identity matrix, and “⊗" is the Kronecker product, whose operation on two arbitrary matrices $G\in R^{k\times \ell }$ and $H\in R^{n\times m}$ produces a matrix $G⊗H\in R^{k\cdot n\times \ell \cdot m}$ such that

(5) $$G⊗H=\left(\begin{array}{ccc} H\;G_{11} & \cdots & H\;G_{1\ell }\\ \vdots & \ddots & \vdots \\ H\;G_{k1} & \cdots & H\;G_{k\ell } \end{array}\right).$$

In summary, the explicit monthly method first solves for ${\hat{m}}_{j}$ from **d**_*j*_ in Equation (1) for *j*=1, . . . , *N_t_* and then solves for $\hat{x}$ from the ${\hat{m}}_{j}$ via Equation 2.

By contrast, the L1B regression mascon products, $\hat{X}$, are solved for directly from the **d***_j_* for *j*=1, . . . , *N_t_*, in a manner similar to

(6) $$\hat{x}={\left[\left(F^{T}⊗I\right)\left(A^{T}WA+P\right)(F⊗I)\right]}^{-1}\left(F^{T}⊗I\right)A^{T}Wd,$$

where **A**, **W**, and **P** are block-diagonal matrices whose *j*^th^ blocks are given by **A***_j_*, **W***_j_*, and **P***_j_*, respectively, and $d^{T}=\left[d_{1}^{T}\ldots d_{j}^{T}\ldots d_{N_{t}}^{T}\right]$. However, in the actual L1B regression case, the term (**F**^T^ ⊗ **I**) **P** (**F** ⊗ **I**) is treated as a diagonal matrix corresponding to a signal covariance that is encoded only as auto-covariant terms in the temporal multipliers with no cross-covariance. Aside from this technicality, it should be clear that Equations (1) and (4) are nested inside Equation (6). In fact, if the formal error-covariances, ${\left(A_{j}^{T}W_{j}A_{j}+P_{j}\right)}^{-1}$, of each estimate ${\hat{m}}_{j}$ from Equation (1) were incorporated into the estimate $\hat{x}$ in Equation (4), then the explicit method would be equivalent to the L1B regression method. However, this is precisely why the L1B regression is superior to the explicit method since it does not ignore the error-covariances on the ${\hat{m}}_{j}$. The explicit method has traded the self-consistent propagation of error in the L1B estimate for the convenience of estimating $\hat{x}$ via simple, independent estimates of each ${\hat{x}}_{k}$ in Equation (2). It turns out that while the explicit method provides an unbiased estimate of $\hat{x}$ in the ideal case (i.e., assuming $\hat{m}$ is unbiased, which Loomis et al. (2019) shows is not the case), it does not provide a minimum-variance solution in contrast to the L1B regression method. Given that nature provides only a single sample of the data, it is far superior to draw $\hat{x}$ from a distribution more narrowly centered on the true value of $\hat{x}$ that is provided by the L1B regression method then from a broader distribution provided by the explicit method. In practice, the enhanced spatial resolution of the L1B regression solution is due to the reduced strength of the regularization applied in Equation (6) than is required for estimating $\hat{m}$ in Equation (1).

#### 2.1.3. Confidence Intervals

The rigorous characterization of uncertainties is critical for the proper interpretation of GRACE TWS estimates. Loomis et al. (2019) demonstrate the importance of properly accounting for the bias (or leakage) of regularized solutions, and provide detailed procedures for building the total GRACE monthly mascon error budget. To frame the issue we begin with the expression that defines the regularized linear least-squares mascon estimate by rewriting Equation (6) as

(7) $$\hat{x}={\left(H^{T}WH+Q\right)}^{-1}H^{T}Wd,$$

where

(8) H=A(F⊗I),

(9) $$Q=\left(F^{T}⊗I\right)P\;(F⊗I),$$

The construction of **Q** is the key design parameter for regularized mascon estimation, and any non-zero **Q** almost certainly introduces a bias in the solution. As previously noted in Hoerl and Kennard (1970) and Kusche and Springer (2017), the solution bias, **b**, is defined by the expected value of the difference between the estimated state, $\hat{x}$, and the unknown true state, **x**:

(10) $$b\equiv E\left[\hat{x}-x\right]=(R-I)\;x,$$

where **R** is termed the model resolution operator and is defined as:

(11) $$R\equiv {\left(H^{T}WH+Q\right)}^{-1}H^{T}WH.$$

Note that the unregularized solution results from setting **Q** to zero, in which case **R** becomes the identity matrix and the bias is zero (this case produces an unusable solution due to its unmitigated noise). The implication of Equation (10) is that a rigorous assessment of the mascon uncertainties must account for the solution bias.

As the true mascon state, **x**, is unknown, some assumptions must be made in order to compute the solution bias described by Equation (10). Following the procedure in Kusche and Springer (2017) and Loomis et al. (2019) substitute $\hat{x}$ for **x** in Equation (10) to define the bias. Alternatively, if one begins with the assumption that some independent data set (or combination of data sets) represents the true signal, the independent data defines **x** and a value for the bias can be computed. In the absence of noise errors, the estimated mascon state resulting from Equation (7) is exactly equal to the resolution operator multiplied by the unknown true state: $\hat{x}=\mathrm{Rx}$ (Menke, 2015; Loomis et al., 2019). Considering this expression and the assumption that the independent data set, **x**_model_, is the truth (i.e., **x**_model_ = **x**), then **Rx**_model_ is contained within $\left[\hat{x}-z\sigma ,\hat{x}+z\sigma \right]$, where *zσ* defines the half-width of the confidence interval (for normally distributed errors, *σ* is the noise standard deviation and *z* = 2 for ~95% confidence). This is the common form of confidence intervals, which are typically reported as $\hat{x}\pm z\sigma$. A direct comparison between **x**_model_ and $\hat{x}\pm z\sigma$ neglects the bias/leakage that is quantified by applying **R**. This concept is similar to the common method of applying the same smoothing or post-processing to both **x**_model_ and $\hat{x}$ to facilitate comparative analyses, which is a reasonable approach when $\hat{x}$ is defined by unregularized spherical harmonics (though the post-processed spherical harmonics have the shortcomings discussed in section 2.1.1).

If we want to form the comparison in terms of the independent data set instead of the GRACE solution, then **x**_model_ is contained by the confidence interval $\left[\hat{x}-b-z\sigma ,\hat{x}-b+z\sigma \right]$ if it is the true signal. The hypothesis **x**_model_ = **x** can be tested for individual mascons or for any combination of mascons that define a basin or region. If **x**_model_ is contained by the interval then the hypothesis is not disproved and the independent data set is considered to be in agreement with GRACE. Conversely, disproving the hypothesis is an effective method for identifying specific mascons and basins where additional data and/or model improvements are needed to close the TWS trend budget. When reporting our GRACE-derived regional trends we report the first type of confidence interval, $\hat{x}\pm 2\sigma$, as this follows common practice and the bias/leakage errors are relatively small for the L1B regression product at regional scales. When testing the hypothesis **x**_model_ = **x** for individual mascons, we consider the 99% confidence interval, $\left[\hat{x}-b-2.576\sigma ,\hat{x}-b+2.576\sigma \right]$, and note that this interval is not guaranteed to contain $\hat{x}$. We test the hypothesis **x**_model_ = **x** for the glacier mass balance and groundwater data sets only, due to their relatively good agreement with the GRACE trends over their respective regions. The noise uncertainties for the high-resolution trends are determined by examining the statistics of the ocean mascons, which are expected to be close to zero, meaning that their spread should approximate the solution noise (similar statistics are observed in the Sahara desert, which is also expected to have near-zero trends). We note that in Loomis et al. (2019) all equations are developed for monthly mascon estimation, while for this work we have extracted the trend-only portion of **R**, which our analysis shows is largely independent of the bias and annual components.

Lastly, we note that the applied GIA model and geocenter corrections are also non-negligible sources of error for regional GRACE mass trend estimates. These errors are insignificant at the mascon level, with maximum magnitudes of ~0.1 cm yr^−1^. To account for these errors the total regional uncertainties reported in Table 1 and Figure 5B are computed as the root-sum-square (RSS) of the 2*σ* noise, the GIA model error, and the geocenter correction error. We define the GIA error as the difference between the A et al. (2013) and ICE-6G_D (Peltier et al., 2018) models, and the geocenter error as the difference between Swenson et al. (2008) and Sun et al. (2016).

### 2.2. Glacier Mass Balance

Glaciers cover approximately 98,000 km^2^ of the HMA region, and their mass is constantly changing in response to accumulation (primarily snowfall), and ablation (primarily surface melt). For this study, we use new 2000–2018 geodetic glacier mass balance observations derived from DEM time series for all 95,536 glaciers in HMA (Shean et al., submitted). The NASA Ames Stereo Pipeline (Shean et al., 2016) was used to process archives of 15 m ASTER stereo imagery and sub-meter DigitalGlobe WorldView-1/2/3, and GeoEye-1 imagery. The observed elevation trend for each glacier was converted to volume change using Randolph Glacier Inventory (RGI) polygons (Consortium, 2017), and mass change for the 2000–2018 period was estimated using standard density values (Huss, 2013). The water equivalent sum of glacier mass balances was calculated for each mascon, enabling direct comparison with mass trends observed by GRACE. The geometric centroid of each glacier polygon was used to assign each glacier to a specific mascon. Noting that the mass balance signal is expected to be larger in mascons with more glaciated area, we report regional trends for both the full glaciated region shown in Figure 1B, and for the subset of mascons with ≥100 km^2^ of glacier area.

### 2.3. Groundwater

#### 2.3.1. Groundwater Level Time Series and Aquifer Properties

Historical depth-to-water (DTW) measurements between 2003 and 2016 have been collated from 9,976 dug wells (unconfined aquifer) and 3,673 tube wells (confined aquifer) from the India Water Resources Information System (NWIC, 2018) and through personal communications with Dr. Tess Russo at Intellectual Ventures and Dr. Naveed Iqbal at Pakistan Council for Research on Water Resources. The distribution of dug wells is widespread across northern India but limited in Pakistan covering only parts of the Punjab Province. The tube well data are available only in India and are geographically restricted to locations with a confined aquifer system. The available data covers the majority of the domain where large and complex aquifers are present (Richts et al., 2011). The vertical datum for the DTW measurements is defined relative to the local land surface elevation. The raw DTW data are pre-processed to remove any negative values, incorrect geographic coordinates, and anomalous DTW values likely resulting from typographical errors.

Storage coefficients are necessary to convert from groundwater level anomalies to groundwater storage anomalies. These coefficients include specific yield for unconfined aquifers and the product of specific storage and aquifer thickness for confined aquifers. Specific yield values are parameterized using percentages of sand, silt and clay (Hengl et al., 2014) that are classified into soil texture class boundaries (Soil Survey Division Staff, 1993) and then assigned specific yield ranges by texture class (Johnson, 1967). Since storage coefficients for confined aquifers storage are rarely available in this region, we use typical values published in the literature from other regions (Domenico and Schwartz, 1997). We also use an upper limit for the storage coefficient in the confined areas equal to the specific yield, i.e., in the event the water levels fall below the top of the confined aquifer, which can occur due to overpumping. We compared trends in the total groundwater storage anomaly with trends in GRACE TWS, both at regional and individual mascon scales, to determine the combination that provides the best agreement under the assumption that the GRACE trend is predominantly driven by groundwater in this region (discussed further in section 3.3). The anomalies for the unconfined and confined portions are combined for calculating the total groundwater storage anomaly across the study domain.

#### 2.3.2. Gridded Groundwater Level Anomalies

Monthly groundwater level anomalies (GWLA) were calculated at each well by removing the study period (2003-2016) mean DTW from observed values such that positive anomalies indicate a rise in water level and vice-versa. GWLA for months with no measurements are calculated using linear interpolation between successive times not more than four months apart. These point GWLA calculations were used to estimate gridded groundwater level anomalies (gridded-GWLA) using the kriging interpolation scheme at 0.25° spatial resolution. The gridded-GWLA for unconfined aquifers is estimated using GWLA calculated from individual dug wells, and gridded-GWLA for confined aquifers is estimated using GWLA calculated from individual tube wells. These gridded-GWLA values are multiplied by the appropriate aquifer storage coefficients to obtain monthly gridded groundwater storage anomalies (gridded-GWSA).

### 2.4. Land Surface Model Outputs From NASA LIS

NASA LIS is a land surface modeling and data assimilation environment that facilitates the use of ensemble land surface modeling with multiple LSMs, meteorological boundary conditions, land surface parameters, and data assimilation options. In order to study terrestrial water budget estimates and their uncertainties, an ensemble of land surface model runs was conducted using a combination of two different LSMs and three different sets of meteorological boundary conditions. The Catchment Land Surface Model (CLSM) version Fortuna 2.5 (Ducharne et al., 2000; Koster et al., 2000) and Noah-MP LSM version 3.6 (Niu et al., 2011; Yang et al., 2011) are forced with meteorological boundary conditions derived from MERRA-2, GDAS, and ECMWF. Note that we chose this subset of boundary conditions as they meet the spatial and temporal coverage needs for this comparison. In addition, we use the CHIRPS2 precipitation product (in conjunction with near surface meteorology derived from ECMWF) because CHIRPS2 is found to have relatively low errors, high correlations, and better consistency of trends in the precipitation evaluations presented in Yoon et al. (2019). The evaluation of the terrestrial water budget from this suite of model runs was found to provide comparable estimates to those reported in global studies such as Rodell et al. (2015).

Even though the LSMs used in this study lack glacier physics (i.e., mass balance) and only account for shallow groundwater, the output from these LSMs is valuable as it serves to fill in the process gaps (in space and time) that is not captured in the observational record. When the LSMs are convolved with the glacier and groundwater estimates derived from stereo imagery and well measurements, respectively, a more cohesive view of terrestrial water storage across HMA is achieved that could not be made using any one of the data products on its own. Furthermore, GRACE-derived TWS retrievals provide an independent evaluation of the integrated stereo imagery (glaciers), well measurement (deep groundwater), and LSM (soil moisture, snow, surface runoff, shallow groundwater) estimates such that an assessment of HMA water balance closure, or lack thereof, may be made.

## 3. RESULTS AND DISCUSSION

### 3.1. GRACE Total Water Storage

GRACE-derived regional mass trends for HMA and the regions defined in Figure 1B are reported in Table 1. Excellent agreement is achieved between the independent GRACE solutions for the full HMA region, which is defined as the combined set of mascons with either glacier mass balance, groundwater, or LIS data. The spread in the GRACE solutions is greater for the smaller regions within HMA, especially those that have significant trends near the regional boundaries. The GSFC L1B regression solution reports notably larger mass losses for the glaciated region, the region sampled by groundwater measurements, and the Brahmaputra basin. These differences can be attributed to the significant reduction in signal leakage achieved by the L1B regression product due to its improved spatial resolution, which is made evident by comparing Figures 2A,B. We specifically highlight the enhanced signal recovery of the mass losses in the Tien Shan mountains (43°N, 86°E), the eastern Himalayas (30°N, 95°E), and northwestern India (28°N, 76°E). The largest trend magnitude within HMA is for the identified mascon in northwestern India, which is −11.7 cm w.e. yr^−1^ for the L1B regression solution and only −4.1 cm w.e. yr^−1^ for the monthly mascons.

Our preferred GRACE L1B regression solution reports a mass trend of −37.8 ± 10.4 Gt yr^−1^ for the full HMA region, where the glaciated region accounts for −23.6 ± 5.5 Gt yr^−1^. As mentioned above, a fairly large spread of GRACE-derived HMA glacier mass trends exists between published results, with previous studies reporting values of −47 ± 12 Gt yr^−1^ (Matsuo and Heki, 2010), −4 ± 20 Gt yr^−1^ (Jacob et al., 2012), −19 ± 20 Gt yr^−1^ (Gardner et al., 2013), and −17.7 ± 11.3 (Wouters et al., 2019). These previous efforts sought to isolate the glacial mass change by removing both the GIA and non-glacial hydrologic components of the trend, while our reported value of −23.6 ± 5.5 Gt yr^−1^ has only removed GIA. The suite of LIS outputs described in section 2.4 yield an average mass trend of −3.1 Gt yr^−1^ for the subset of LIS/glaciated mascons, and we note that our LIS region encompasses most but not all of the glaciated region. If we remove this mean hydrologic trend from our preferred GRACE estimate, the glacier mass loss becomes −20.5 Gt yr^−1^, which agrees with the more recent assessments of Gardner et al. (2013) and Wouters et al. (2019). We note that both of these previous studies apply a complex set of post-processing procedures to the Level 2 GRACE data sets in an effort to mitigate signal leakage, while our preferred trend solution has been directly estimated from the Level 1B measurements resulting in the improved signal recovery shown in Figure 2.

Below we discuss TWS mass trends in the context of our effort to close the budget between the GRACE-derived values and the independent glacier mass balance, groundwater, and LIS data sets at both regional and mascon spatial scales. The mascon-level comparisons apply our novel approach to build rigorous confidence intervals by employing the resolution operator in the computation of the solution bias as detailed in section 2.1.3. While previous studies implicitly close the water budget by using a residual to isolate water budget components or fully assimilating GRACE into land surface models, we demonstrate where further work is needed to close the budget by leveraging the availability of independent glacier mass balance and groundwater data.

### 3.2. Glacier Mass Balance

The total mass balance of HMA glaciers during 2000–2018 derived from geodetic observations is −19.0 ± 2.3 Gt yr^−1^ Shean et al. (submitted), which agrees well with the −16.3 ± 3.5 Gt yr^−1^ estimate by Brun et al. (2017) during 2000–2016. The 2003–2016 GRACE GSFC L1B regression estimate (after removing GIA only) is −23.6 ± 5.5 Gt yr^−1^ The successful closure between the new geodetic and GRACE mass trends over the glaciated region demonstrates that the GRACE-observed mass trends are largely dominated by glacier mass balance. If we limit the considered region to mascons with at least 100 km^2^ of glacier area the agreement further improves, with a geodetic glacier mass balance estimate of −18.0 Gt yr^−1^ and GRACE estimate of −15.9 ± 3.6 Gt yr^−1^. We also note that the agreement between the geodetic and GRACE values is vastly improved for the L1B regression estimate as compared to the monthly mascon products.

**FIGURE 3 ∣:**
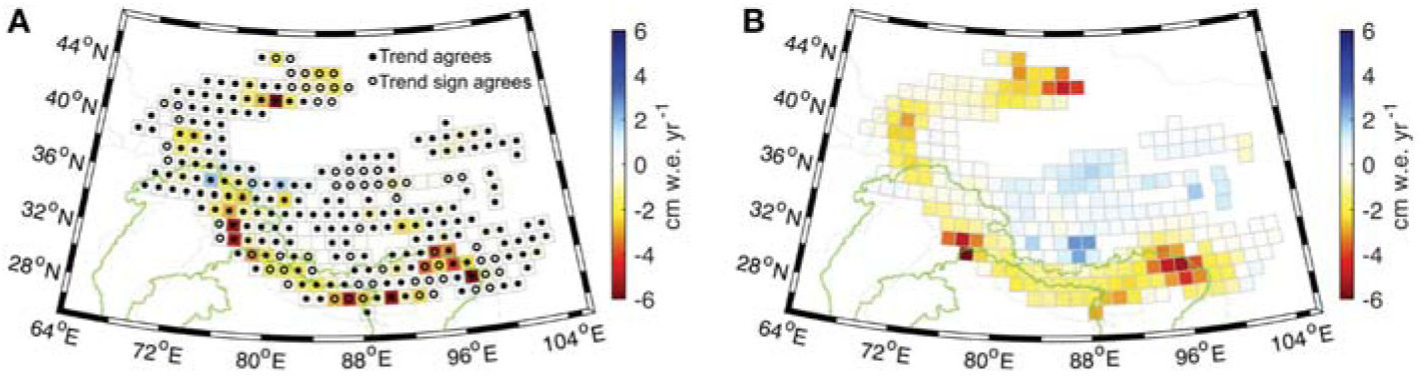
Comparison between mass trends for **(A)** geodetic glacier mass balance for 2000–2018 and **(B)** GRACE L1B regression mascons for January 2003–July 2016. The open circles in **(A)** indicate where the sign of the glacier mass balance trend agrees with the 99% confidence interval and the closed circles indicate where the glacier mass balance trend estimates are within the 99% confidence interval.

Following section 2.1.3, we assess mass budget closure in the glaciated region between the geodetic mass balances and GRACE for individual mascons by accounting for the solution bias/leakage via the resolution operator. Over the full glaciated HMA region, we find that the glacier mass balances in 72% of individual mascons lie within the 99% GRACE-derived confidence intervals, while the sign of the mass balance agrees with the confidence intervals for 96% of mascons (Figure 3). The greatest disagreements between GRACE and glacier mass balance trend exist over the eastern Himalaya (24-30°N, 92-100°E) and the Pamir and Tien Shan mountains (40-44°N, 80-88°E). These regions generally correspond with areas of reduced density of DEMs available for the elevation change analysis, and hence tended to have larger uncertainties. We also observe some disagreement between GRACE and geodetic mass balances over the inner Tibetan Plateau (30-36°N, 80-92°E), with GRACE showing some areas of mass gain not apparent in the glacier datasets. Satellite altimetry and lake area data suggest lakes on the Tibetan Plateau have been increasing in volume during 1990–2015 (Treichler et al., 2018). These lake changes may account for some of the observed mass increase not attributed to our glacier observations, however most of the lake volume increase is associated with a step-like increase in precipitation in 2000 that pre-dates the GRACE observation period.

Our analysis supports the use of GRACE data to independently assess the long-term mass trends of glaciers in the HMA region, using the resolution operator approach described in this paper. While our findings suggest that glacier processes dominate the long-term water budget for regions where glaciers are located, they do not necessarily support the use of standard monthly GRACE solutions to represent year-to-year or seasonal glacier mass balances. This is because leakage and attenuation of signal have a much larger impact on monthly GRACE solutions than our approach to directly estimate mass trends from the L1B data, which is designed to maximize spatial resolution over the full GRACE record. In addition, fully accounting for sub-annual glacier mass balance requires additional corrections for, among other factors, seasonal accumulation and ablation of snow on non-glacier land surfaces.

### 3.3. Groundwater

**FIGURE 4 ∣:**
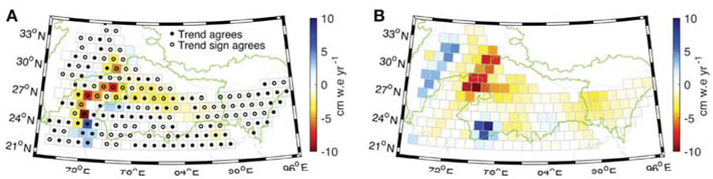
Comparison between mass trends for **(A)** groundwater data and **(B)** GRACE L1B regression mascons. The open circles in **(A)** indicate where the sign of the groundwater trend agrees with the 99% confidence interval and the closed circles indicate where the groundwater trend estimate is within the 99% confidence interval. Trends are computed for January 2003–July 2016.

The regional GRACE trend is partially explained by the trend in groundwater in the study domain across northern India, where *in situ* water levels are available, as shown in Figure 4. Considering the set of mascons where groundwater data is available (Figure 1B), the GRACE trend is −23.2 ± 4.3 Gt yr^−1^. The total groundwater trend is 1.35 Gt yr^−1^ (2 Gt yr^−1^ for unconfined, −0.64 Gt yr^−1^ for confined) when it is assumed the confined areas are acting as fully confined layers, and becomes −13.6 Gt yr^−1^ (2 Gt yr^−1^ for unconfined, −15.6 Gt yr^−1^ for confined) when it is assumed that the confined water levels are behaving in an unconfined manner. The latter situation results in a trend that is similar to other studies in the region that use wells from the same database as ours and only apply specific yield to all study wells (MacDonald et al., 2016; Mukherjee et al., 2018), and we believe this is a reasonable approach because the seasonality of the water levels in both the confined and unconfined aquifers are very similar suggesting a connection along with the possibility that dropping water levels over time below top of the confined aquifer could result in unconfining conditions. This approach is further supported by the closer match to the GRACE trend, along with previous literature explaining the trend is due to groundwater declines. We report gridded-GWSA trends of +2.0 Gt yr^−1^ for the unconfined and −0.64 Gt yr^−1^ to −15.6 Gt yr^−1^ for the confined layers, depending on the storage coefficient used. MacDonald et al. (2016) found a stable or increasing trend in 70% of the Indo-Gangetic Basin. Their study corroborates our findings between both the GRACE trend and the *in situ* groundwater trend of increases in water storage along the Indus River and across the northern boundary of our groundwater domain, as well as declining trends in northwestern India. Significant groundwater depletion has been well documented in northwestern India, including in the states of Rajasthan, Haryana, and Punjab (Rodell et al., 2009; Tiwari et al., 2009), and this region is known for extensively irrigated agriculture (Zaveri et al., 2016).

**FIGURE 5 ∣:**
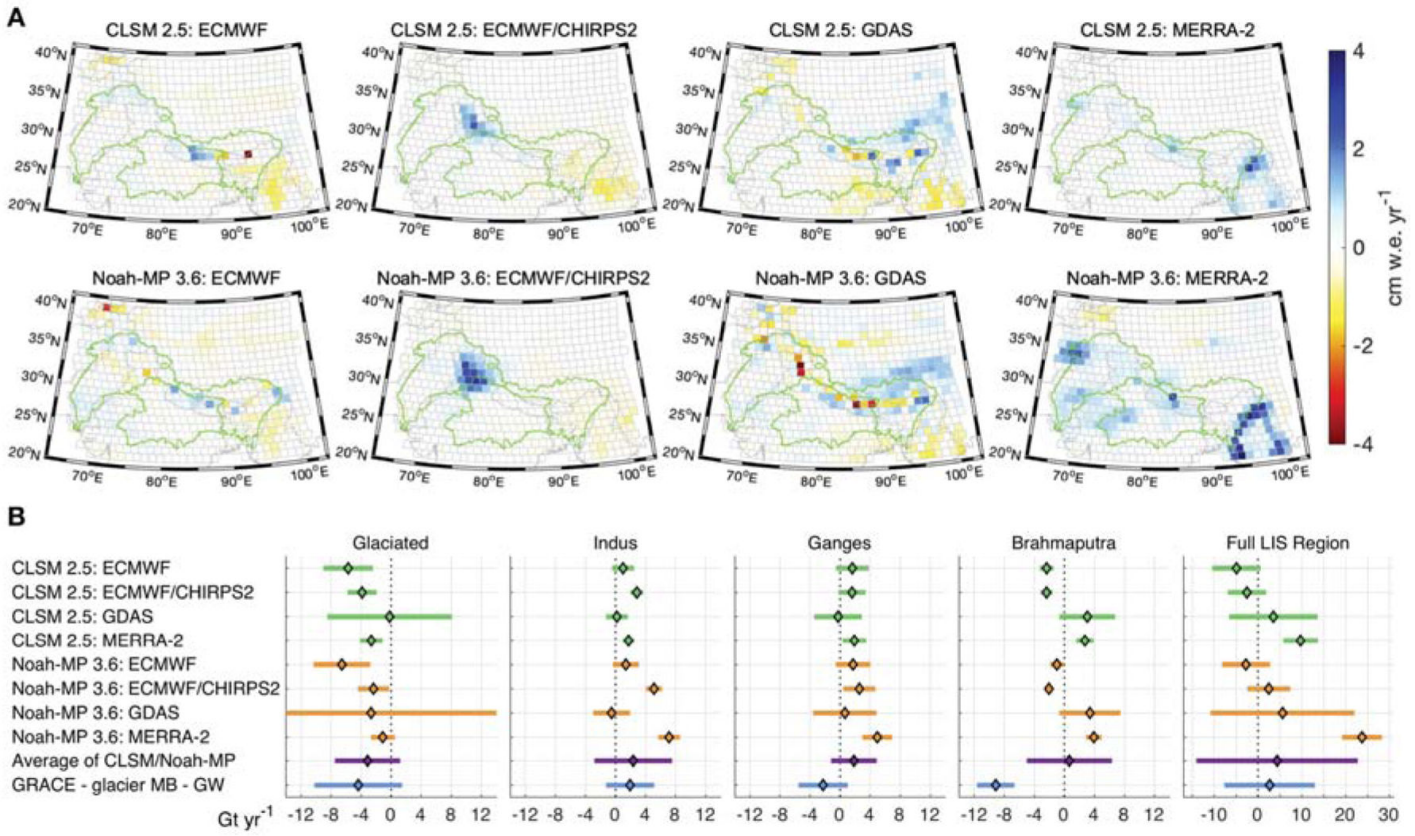
**(A)** Trend maps for the eight different LIS outputs. **(B)** Summary of regional trend values for LIS outputs and GRACE with the glacier mass balance and groundwater signals removed (see Figure 1B for region definitions). Trends are computed for 2004–2016 to match the available LIS data. The CLSM/Noah-MP uncertainties represent 2*σ* noise, the “Average of CLSM/Noah-MP” errors are twice the standard deviation of the eight CLSM/Noah-MP values, and the uncertainties for “GRACE - glacier MB - GW” include the 2*σ* GRACE noise, GIA model error, and geocenter correction error.

Figure 4 shows that the trend sign agrees in almost every mascon in northwestern India, however the magnitude of the trend is generally smaller for the groundwater observations than for GRACE. The LIS outputs in this region (Figure 5), which is not glaciated, varies in both sign and magnitude. The majority of pumping in this domain is from confined layers (Panda and Wahr, 2016), however approximately two-thirds of the wells used in this study within the confined area (based on maps from Kumar et al., 2012) are unconfined. Therefore, part of the discrepancy between the GRACE trend and the observed groundwater trend in this region could potentially be due to an under-representation of confined wells. The majority of GRACE studies that attribute the high water loss rates to deep groundwater use TWS model output to isolate the groundwater residual, though the models neglect anthropogenic impacts such as irrigation and diversions through canals. It is possible that some of the discrepancy is also driven by changes in other components of the water budget that are not robustly represented in the models, especially those impacted by human dynamics. The results in the southwestern and central portions of the groundwater domain show an increasing trend from both the groundwater levels and GRACE. It has previously been shown that the region with the highest increasing trend over the state of Gujarat has increasing water levels due to policy changes that decreased groundwater pumping (Bhanja et al., 2017). An increasing trend in precipitation in the central region subsequently increases recharge into the unconfined system, causing the positive trend (Rodell et al., 2018). This study demonstrates that further work is needed to close the water budget in this domain and that the total groundwater trends in the region are largely driven by the confined aquifers, indicating that the anthropogenic influence of groundwater pumping is a key driver of TWS change across this region.

The utility of groundwater levels to validate GRACE-derived groundwater estimates is challenged by the need for groundwater storage coefficients to convert from water levels to storage changes, which tend to be sparse if available at all. Previous studies have used GRACE to constrain model parameters related to soils and groundwater (Lo et al., 2010; Sun et al., 2012), however, they emphasize unconfined parameters. Here, we use prior knowledge from the literature, and the demonstration of the relatively small influence of the LIS TWS components over most of the region, to assume that the GRACE trend is largely driven by deep groundwater storage changes. This assumption allows us to tune the storage coefficient used to convert water levels to storage anomalies. The results are promising given our analysis which shows that the majority of the regional GRACE trend is accounted for by the groundwater estimates over the relevant region. Further work, however, is needed to continue to refine the groundwater parameters given that only 44% of the mascons agree to within the 99% confidence interval. This indicates that either the storage coefficients are not yet representative of true values at the mascon scale and/or that the declining trend from groundwater is being partially offset by an increasing trend in a different water storage component that is integrated into the GRACE trend.

### 3.4. Terrestrial Water Storage via Land Surface Models

Figure 5A presents the trend maps of each combination of LSM and meteorological boundary condition tested within LIS, while Figure 5B compares the various regional LIS trends and their average value to those obtained by removing the glacier mass balance and groundwater from the GRACE L1B regression trend estimates (referred to hereafter as “GRACE-corrected”). Clearly the spatial structure of the LIS output is highly dependent on the selected meteorological forcing. When considering the average of the LIS results at regional scales and solution uncertainties, trend budget closure is achieved for the glaciated region, the Indus and Ganges basins, and the full LIS simulation region (following the region definitions in Figure 1B). The LIS simulations, regardless of the LSM or meteorological boundary conditions used, suggest a small gain within the Ganges basin (average value of 1.8 ± 3.1 Gt yr^−1^), while the GRACE-corrected value reports a small mass loss (−2.3 ± 3.3 Gt yr^−1^), though agreement is achieved when accounting for uncertainties. In the Indus, we observe a larger spread in the LIS results, but report excellent agreement between their average value (2.4 ± 5.2 Gt yr^−1^) and the GRACE-corrected trend estimate (1.9 ± 3.2 Gt yr^−1^). Comparing over the full LIS simulation domain, the GRACE-corrected trend (+2.6 ± 10.3 Gt yr^−1^) is encapsulated by the spread of both the LSMs, where Noah-MP trends range from −4 to +24 Gt yr^−1^, CLSM yields trends of −5 to +10 Gt yr^−1^, and the mean of all LIS outputs is +4.3 ± 18.4 Gt yr^−1^.

Budget closure is not achieved for the Brahmaptra basin, for which the GRACE-corrected value is strongly negative (−9.1 ± 2.5 Gt yr^−1^), whereas the LIS trends range from weakly negative to weakly positive depending on which precipitation product is applied at the boundary conditions, and have an average value near zero (0.7 ± 5.7 Gt yr^−1^). A likely reason for this behavior is the larger inconsistencies in the precipitation trends over the eastern parts of HMA. For the Brahmaputra the application of ECMWF- or CHIRPS-based precipitation yields a relatively small, negative trend in TWS while GDAS and MERRA-2 precipitation products yield a small, positive trend. As detailed in Yoon et al. (2019), the trend of increasing precipitation in datasets such as GDAS and MERRA-2 is inconsistent with the reported declining trends in precipitation over this area. Other possible reasons for the lack of budget closure here include model structure errors within the LSMs used in this study or the limitations in the regional trends estimated with sparse groundwater observations and the lack of groundwater data in the portion of the Brahmaputra that is outside of India.

Overall, the reported regional GRACE-only trends in Table 1 and the GRACE-corrected trends in Figure 5 reveal that the trends derived from a LSM are insufficient to explain those observed by GRACE. The LSM-based estimates used here primarily reflect the influence of precipitation as a first-order control on the conservation of mass balance at the land surface. The LSMs used in this study have a limited representation of shallow (i.e., unconfined) groundwater dynamics while completely lacking confined groundwater dynamics and the glacier physics needed to compute glacier mass balance. In addition, they do not incorporate the impacts of human management such as agricultural irrigation, groundwater abstraction, and canal diversions. On the other hand, implicit in the GRACE-derived estimates are the changes in the stores of freshwater, including confined groundwater, unconfined groundwater, surface water impoundments, soil moisture, snow, and glacier ice. Therefore, the analysis presented here clearly indicates that the differences between GRACE-derived TWS trends and TWS derived from a land surface model in HMA are more generally related to glaciers and/or confined groundwater.

The observed differences between the LSMs can be attributed to model structural differences. For example, CLSM represents subsurface storage changes from which unconfined groundwater changes can be inferred, and Noah-MP has an explicit unconfined groundwater layer, while neither model represents confined groundwater. The impact of these differences is highlighted in northwestern India in Figure 5A for the ECMWF/CHIRPS2 output for each model. The larger positive anomaly in Noah-MP could be explained by the availability of additional storage as unconfined groundwater to hold precipitation, whereas the subsurface component in CLSM is more tightly connected to the surface layer and has a decreased ability to retain moisture in the subsurface. Previous studies in this domain have compared GRACE-derived groundwater trends to *in situ* observations, finding good agreement in seasonality, but not always a high correlation between trends such as in the Ganges Basin within India (Bhanja et al., 2016). Mukherjee et al. (2018) attribute recent drying trends in the Ganges river to groundwater depletion that is causing a reduction in baseflow. Groundwater abstractions can also lead to an increase in recharge across the Indo-Gangetic Basin (MacDonald et al., 2016), which, when combined with decreases in baseflow, minimize storage loss from the aquifers and instead cause declines in surface water supplies. These human-driven groundwater-surface water interactions are not represented in the LSMs and could explain some of the disagreement between the LSMs and the expected output.

## 4. CONCLUSIONS

We have presented HMA regional mass trends for January 2003–July 2016 as computed from four different GRACE mascon products. The GSFC, JPL, and CSR monthly solution trends range from −34.1 to −36.3 Gt yr^−1^, while the new high-resolution GSFC L1B regression trend product reports a trend of −37.8 ± 10.4 Gt yr^−1^. The good agreement between the GRACE mascon products is an important achievement considering the large discrepancy between previously-published GRACE HMA trends (e.g., Matsuo and Heki, 2010; Jacob et al., 2012), and the significant differences in processing and regularization strategies employed for the four different products. We attribute this improved agreement to the quality of the mascon solutions and the extension of the GRACE data time series, and the larger mass losses of the L1B regression are explained by its improved spatial resolution and the corresponding reduction in signal leakage. The L1B regression mascon HMA mass trend is equivalent to a +0.10 ± 0.02 mm yr^−1^ contribution to global sea level rise, assuming that all HMA mass losses have entered the ocean.

The total mass trends for the geodetic glacier mass balance and groundwater observations are −19.0 Gt yr^−1^ and −13.6 Gt yr^−1^ over their respective sub-regions within HMA. Summing these values results in a combined mass trend of −32.6 Gt yr^−1^, matching our preferred GSFC L1B regression mascon value of −37.8 ± 10.4 Gt yr^−1^ for all HMA within uncertainties. Removing the glacier mass balance and groundwater components from the GRACE trends identifies the combinations of LSM and meteorological boundary conditions that best close the TWS trend budget over the HMA LIS region, and the Indus and Ganges basins, while reporting lack of closure in the Brahmaputra for all combinations. In Figures 3, 4 we have applied a rigorous uncertainty analysis that employs the resolution operator to quantify solution bias in order to determine where TWS trend budget closure has and has not been achieved for each individual ~110 km mascon, identifying geographic regions where additional data and/or model improvements are needed. Investigating the TWS trend budget at these scales is made possible by the new GSFC L1B regression mascon product, which contains higher spatial resolution information than the monthly mascons and improves the signal recovery as compared to previous multi-year spherical harmonic trend estimates.

We have clearly established the significant impact of confined groundwater changes on the HMA TWS trend. Consistent with previous studies, we find that the trend in unconfined groundwater alone is positive (+2.0 Gt yr^−1^) and well outside the uncertainty range of the GRACE trend, but when combined with the confined trend results in a trend of −13.6 Gt yr^−1^. Though agreement with GRACE in this region is not achieved, it is clear that the confined groundwater is a significant contributor to the GRACE-derived trend in HMA. The method used herein to constrain aquifer storage parameters is promising based on the overall regional match between the GRACE L1B regression trend and groundwater across the region. However, further work is required to refine the storage parameters to improve the match at the mascon scale. The lack of agreement between the GRACE and LSM trends in HMA can be largely explained by the missing glacier mass balance and confined groundwater representation in the LSMs, where the groundwater component is lacking both the dynamics and anthropogenic impacts such as pumping. Future work to improve the performance of LSMs in HMA should include the assimilation of the available unconfined groundwater changes, groundwater and surface water abstractions, and an explicit representation of unconfined (CLSM) and confined (CLSM and Noah-MP) groundwater dynamics. Such an effort should significantly improve the LSM agreement with the GRACE-observed TWS trends in HMA.

## Figures and Tables

**TABLE 1 ∣:** GRACE regional mass trends and uncertainties (Gt yr^−1^) for different mascon solutions over the span January 2003–July 2016, along with the applied GIA correction for each region.

Region	GRACE global mascon solution after GIA correction				GIA
	GSFC L1B	GSFC monthly	JPL monthly	CSR monthly	
	[Gt yr^−1^]	[Gt yr^−1^]	[Gt yr^−1^]	[Gt yr^−1^]	[Gt yr^−1^]
Glaciated	−23.6 ± 5.5	−9.9 ± 5.8	−17.8 ± 5.9	−13.9 ± 5.7	7.6 ± 3.1
Glaciated≥100 km^2†^	−15.9 ± 3.6	−6.9 ± 3.5	−12.8 ± 3.7	−8.5 ± 3.4	3.9 ± 1.6
Groundwater	−23.2 ± 4.3	−19.4 ± 13.6	−22.3 ± 13.4	−20.8 ± 13.3	4.9 ± 1.7
Indus basin	−3.9 ± 3.0	−2.9 ± 2.6	−2.6 ± 2.7	−5.2 ± 2.6	2.8 ± 1.1
Ganges basin	−15.1 ± 3.1	−13.3 ± 8.1	−18.0 ± 8.4	−14.6 ± 7.5	2.5 ± 0.8
Brahmaputra basin	−15.6 ± 2.4	−11.4 ± 5.0	−12.8 ± 5.6	−10.8 ± 5.3	2.1 ± 0.7
LIS	−30.1 ± 9.6	−14.3 ± 21.9	−30.3 ± 23.0	−30.0 ± 22.5	16.1 ± 6.2
HMA^‡^	−37.8 ± 10.4	−18.5 ± 21.8	−34.8 ± 23.0	−34.1 ± 22.4	17.5 ± 6.8

The regions are defined in Figure 1B. All reported GRACE mass trends have had the ICE-6G_D GIA model removed. Total uncertainties include the 2σ noise, errors in the GIA model, and the geocenter correction.

† The subset of glaciated mascons that contain at least 100 km^2^ of glacier area.

‡ The combined set of mascons with either glacier mass balance, groundater, or LIS data.
